# Theoretical and Experimental Studies of New Modified Isoflavonoids as Potential Inhibitors of Topoisomerase I from *Plasmodium falciparum*

**DOI:** 10.1371/journal.pone.0091191

**Published:** 2014-03-20

**Authors:** Wilian A. Cortopassi, Julia Penna-Coutinho, Anna C. C. Aguiar, André S. Pimentel, Camilla D. Buarque, Paulo R. R. Costa, Bruna R. M. Alves, Tanos C. C. França, Antoniana U. Krettli

**Affiliations:** 1 Departamento de Química, Pontifícia Universidade Católica do Rio de Janeiro, Rio de Janeiro, Brazil; 2 Laboratório de Modelagem Aplicada a Defesa Química e Biológica, Instituto Militar de Engenharia, Rio de Janeiro, Brazil; 3 Laboratório de Malária, Centro de Pesquisas René Rachou, Fundação Instituto Oswaldo Cruz, Belo Horizonte, Brazil; 4 Faculdade de Medicina, Universidade Federal de Minas Gerais, Belo Horizonte, Brazil; 5 Laboratório de Química Bioorgânica, Núcleo de Pesquisas de Produtos Naturais, Rio de Janeiro, Brazil; Agency for Science, Technology and Research - Singapore Immunology Network, Singapore

## Abstract

DNA topoisomerase I from *Plasmodium falciparum* (*Pf*TopoI), a potential selective target for chemotherapy and drug development against malaria, is used here, together with human Topo I (*Hss*TopoI), for docking, molecular dynamics (MD) studies and experimental assays. Six synthetic isoflavonoid derivatives and the known *Pf*TopoI inhibitors camptothecin and topotecan were evaluated in parallel. Theoretical results suggest that these compounds dock in the binding site of camptothecin and topotecan inside both enzymes and that LQB223 binds selectively in *Pf*TopoI. *In vitro* tests against *P. falciparum* blood parasites corroborated the theoretical findings. The selectivity index (SI) of LQB223 ≥98 suggests that this molecule is the most promising in the group of compounds tested. *In vivo* experiments in mice infected with *P. berghei* showed that LQB223 has an antimalarial activity similar to that of chloroquine.

## Introduction

Malaria is the most lethal parasitic disease, causing 219 million cases and 660,000 deaths annually, mainly in African sub-Saharan countries [1]. Brazil registered 306,000 cases of malaria in 2009, most of which were in the Amazon Region and caused by *Plasmodium vivax* followed by *P. falciparum* [2]. This species may cause severe malaria and death in untreated individuals, especially children under five [3], in addition, it quickly develops resistance under selective drug pressure [4]. Presently, *P. falciparum* is resistant to most available drugs; its low susceptibility to artemisinin-combined therapy (ACT) is also registered, especially in Southeast Asia countries [5]. *P. vivax*, highly prevalent worldwide, now shows resistance to chloroquine (CQ) in several countries, including in Brazil [2], [6]. There is no effective vaccine available against malaria [7], [8], thus the disease control relies on individual protection against the mosquito vector bites, and specific drug chemotherapy. Continuous efforts to develop new antimalarials and other control measures are required. The search for better treatments based on new molecular targets should broaden the therapeutic arsenal and allow strategies to fight drug resistance in human malaria [9]–[11].

**Figure 1:**
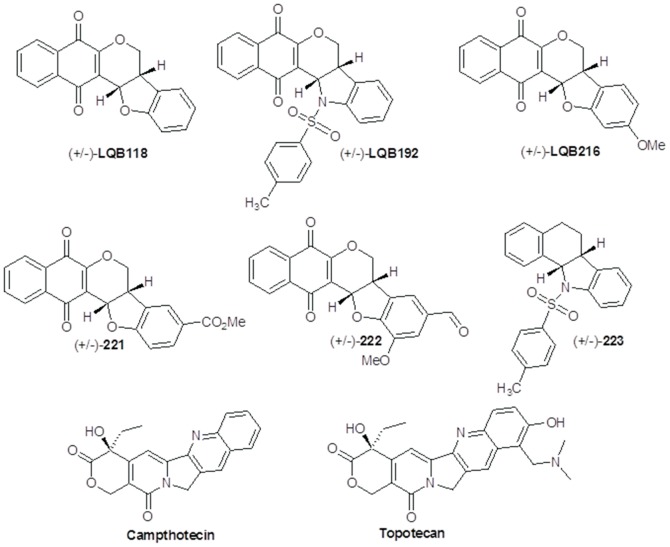
Chemical structures of the new modified isoflavonoids (LQBs), camptothecin and topotecan (Topo I inhibitors).

DNA topoisomerases (Topo) are enzymes involved in DNA replication, transcription, recombination and repair. Although the human Topo I (*Hss*TopoI) presents 42% of similarity with *P. falciparum* Topo I (*Pf*TopoI) [11], this enzyme may represent a potential selective target to be explored for drug development against malaria, the aim of our present work. We used docking, molecular dynamics and experimental assays with six synthetic isoflavonoid derivatives, shown in Figure 1 [12]–[14], in comparison to the known Topo I inhibitors camptothecin and topotecan [15]–[17]. Four are pterocarpanquinones (LQB118, LQB216, LQB221, and LQB222), one is N-tosyl-azapterocarpanquinone (LQB192), and one is N-tosyl-azapterocarpan (LQB223). The results show that LQB223 was the most promising compound, thus, a potential *Pf*TopoI inhibitor and promising new antimalarial lead compound.

## Methodology

### Docking and molecular dynamics simulations

The 3D structures of the compounds in Figure 1 were built and optimized using the Gaussian 03W package [18], and the initial geometries optimized by the method DFT/B3LYP/6-31G [18], [19]. The 3D structure of *Hss*TopoI in complex with topotecan and a 22 base pair DNA duplex used here is available in the Protein Data Bank (PDB) [20] under the code 1K4T [17]. The 3D structure of *Pf*TopoI (Figure S1) was built in the Swiss Model server [21], according to the procedure reported by Roy *et al* [16], using as templates the PDB structures of *Hss*TopoI 1K4T [17] and 1A36 [22]. The coordinates of crystallographic water molecules, topotecan and DNA from 1K4T were copied into the model to obtain the *Pf*TopoI-DNA covalent complex for the docking study.

The docking energies were calculated using the software Molegro Virtual Docker® (MVD) [23]. Our studies were performed considering the covalent complex. Calculations were carried inside a constraint sphere of radius 12 Å with the topotecan as the center, considering the residues within a radius of 15 Å from the center of the sphere as flexible. For the other residues, the rigid docking methodology was performed. Due to the stochastic nature of the docking algorithm, about 20 runs were carried out for each compound, and, 30 poses were found by analyzing the overlap and interactions between ligand and protein. The docking protocol used was previously validated by re-docking topotecan into its own crystallographic structure inside *Hss*TopoI. The best poses of LQB223, based on the lowest values of MolDock Score [23], obtained from the docking studies inside *Hss*TopoI and *Pf*TopoI and complexed with DNA were submitted to additional steps of MD simulations, carried out using the GROMACS 4.5.3 package with the AMBER force field 99SB [24]. The system to be simulated was put into a space-filling box, which was replicated periodically in the x, y and z directions. These systems were studied using the single-point charge (SPC) water model [25]. The minimization algorithms used were performed according to a protocol previously published [26]. The minimized complexes were then submitted to MD simulations for 15 ns using a NpT ensemble, following the same protocol [26]. All Arg and Lys residues were assigned with positive charges and, the residues Glu and Asp were assigned with negative. The short-range Lennard-Jones energies (LJ-SR) and Coulombic potentials within R-coulomb (Coul-SR) were calculated using the g_energy program available in Gromacs, extracting information from .edr files generated during the MD simulations.

### Continuous cultures of *P. falciparum* and antiplasmodial tests

A CQ-resistant and mefloquine-sensitive *P. falciparum*, clone W2 [27] and a CQ-sensitive strain 3D7, were cultivated as described [28], in human erythrocytes (A^+^), in complete medium (RPMI 1640 supplemented with 10% human sera blood group A^+^) and tests followed a protocol [29] slightly modified as follows. Human erythrocytes (A^+^) and serum blood group A^+^ were kindly donated by Center of Hemotherapy and Hemathology of Minas Gerais (HEMOMINAS - http://www.hemominas.mg.gov.br), under the mutual cooperation term number 18/09.

The ring-stage parasites sorbitol-synchronized [30] were adjusted for parasitemia and hematocrit, according to the specifications for the test, then distributed in 96-well microtiter plates (Corning, Santa Clara, CA, EUA), previously prepared and containing the diluted compounds in dimethyl sulfoxide (DMSO) (0.02%) aqueous stock solution, in triplicates for each dose. CQ, the standard antimalarial, was tested in parallel each time. The drug activity was determined in relation to parasite cultures in complete medium and no drugs, as described [31]. Parasite growth was measured through the anti-HRPII test [32] in cultures adjusted to 1.5% hematocrit and 0.05% parasitemia, with monoclonal antibodies (MPFM-55A and MPFG-55P) commercially acquired (ICLLAB®, USA) and the TMB chromogen (3,3′,5,5′-Tetramethylbenzidine) from KPL (Gaithersburg, MD, EUA). The reaction was stopped with 50 µL/L of 1 M sulfuric acid, absorbance read at 450 nm in a spectrophotometer (SpectraMax340PC384, Molecular Devices). Sigmoid dose-response curves were generated with curve-fitting software (Microcal Origin Software 5.0, Inc.), used to determine the 50% inhibitory concentration of the parasite growth (IC_50_), and activity calculated by comparing growth in drug-exposed cultures and the drug-free control cultures.

### Cytotoxicity tests

The cytotoxicity tests were performed against normal kidney glomerular cells from green monkey (BGM) as follows. The cells were cultured under a 5% CO_2_ atmosphere, at 37°C, in 75 cm^2^ sterile flasks in RPMI 1640 medium supplemented with 10% heat-inactivated fetal calf serum, and 40 mg/L gentamicin. When confluent, the cell monolayer was washed with culture medium, trypsinized, distributed in flat-bottomed 96-well plates (5×10^3^ cells/well), and incubated for 18 h at 37°C for cell adherence [33]. The compounds (20 µL) were added to the cell plates, at various concentrations (1000 to1 µg/mL), incubated for 24 h, then received a solution of MTT [3-(4,5-dimethylthiazol-2-yl)-2,5-diphenyltetrazolium bromide] (5 mg/mL; 20 µL/well), followed by another 3 h incubation, to evaluate mitochondrial viability. The supernatants were removed, DMSO added to each well (100 µL), carefully mixed to solubilize the formazan crystals. The optical density was determined at 570 and 630 nm (background) (SpectraMax340PC384, Molecular Devices). Cell viability was expressed as the percentage of the control absorbance in the untreated cells after subtracting the appropriate background. The minimum lethal dose for 50% of the cells (MLD_50_) was determined [34] and the selectivity index (SI) calculated as the ratio between cytotoxicity and activity [35].

### Antimalarial tests against *P. berghei* in mice

The use of laboratory animals was approved by the Ethics Committee for Animal Use of the Oswaldo Cruz Foundation - Fiocruz (CEUA LW-23/13), being in accordance with the principles of the Brazilian Society of Laboratory Animal Science (SBCAL - http://www.cobea.org.br/).

The antimalarial suppressive test in mice, as previously described [36], was slightly modified [37]. Briefly, Swiss outbred adult female mice, 20±2 g weight, were inoculated with *P. berghei* CQ-sensitive (NK65) by intraperitoneal route, 1×10^5^ freshly infected red blood cells (iRBC) per animal. Two to 24 h later, the mice were randomly distributed to be submitted to drug treatment by oral route, during three consecutive days, once a day; two control groups were used, one non treated and another treated with CQ (either 5 or 15 mg/kg).

The molecule LQB223, freshly diluted with 3% DMSO in water immediately before treatment, was given by gavage, in doses of 100 and 50 mg/kg in 200 µL volume per animal. Blood smears were prepared at days fifth and seventh after parasite inoculation, methanol-fixed, stained with Giemsa, and examined microscopically for parasitemia determination. The inhibition of parasite growth (IPG) in the treated groups was evaluated by comparison with the non-treated mice, through the equation: IPG = 100 minus the mean parasitemia in the treated mice, multiplied by 100, and divided by the mean parasitemia in non-treated controls. Molecules reducing parasitemia by 30% or more were considered active.

## Results

### Docking

**Figure 2:**
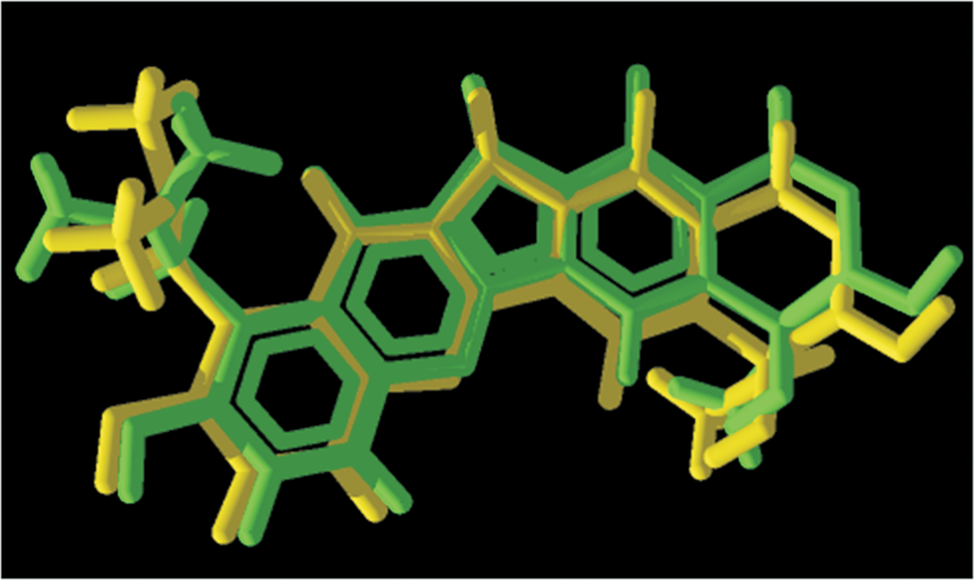
Superposition of topotecan (yellow) to its crystallographic structure (green) after re-docking calculations.

The re-docking calculations showed superposition of topotecan to its crystallographic structure (Figure 2) with a Random Mean Square Deviation (RMSD) of 0.54 Å which, according to literature, is acceptable [19], [38], [39]. LQB223 presented the most comparable docking values to camptothecin and topotecan (Table S1), thus the most promising and selective inhibitor for *Pf*TopoI. The results suggest that LQB223 may establish better interactions with *Pf*TopoI (−187.5 cal mol^−1^) than with *Hss*TopoI (−171.2 Kcal mol^−1^). The main residues involved in the interactions with LQB223 (Table S1) were different for *Hss*TopoI (Ans352, Glu356, Arg364, Tyr452 and Lys425) and *Pf*TopoI (Arg312, Lys512, Asp513, Gln698 and Thr701).

### In vitro and in vivo studies against malaria parasites

**Figure 3:**
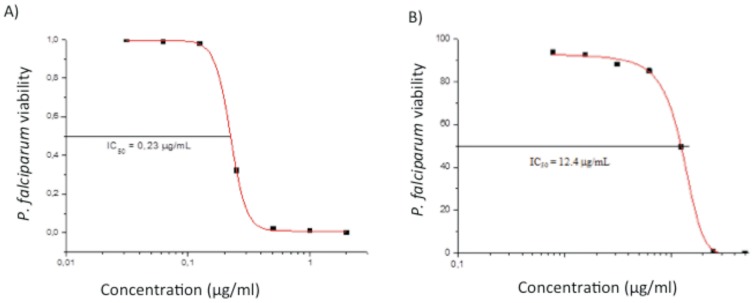
Dose response effect of LQB216 (A) and LQB223 (B), tested in parallel against the CQ-resistant *P. falciparum*.

The assays of drug activity were performed in three independent experiments, using *P. falciparum* iRBC with parasites CQ-resistant (W2 clone) and CQ-sensitive (3D7 strain), in the anti-HRPII test. All topoisomerase I inhibitors were active in micromolar range (Table 1). The most active compounds were LQB118 and LQB216 (IC_50_ 0.17 and 0.4 µg/ml for W2, and, 0.16 and 0.21 for 3D7 parasites); nevertheless, LQB223 was less active against both, the 3D7 (IC_50_ = 7.8±3 µg/mL), and W2 parasites (IC_50_ = 10.2±1.2 µg/mL). The IC_50_ values for CQ were 70 ng/ml (W2 clone) and 3.5 ng/ml (3D7 strain) in our previous work [9], [10]; these parasite susceptibilities to CQ were herein confirmed in parallel experiments (Table 1). The growth inhibition curves and the IC_50_ values for molecules LQB216 and LQB223, against the CQ-resistant *P. falciparum*, are illustrated in Figure 3.

**Table 1:** *In vitro* activities (IC_50_) of Topo I inhibitors determined in assays against *P. falciparum* blood parasites, CQ-sensitive (3D7) or CQ-resistant (W2), and cytotoxicity against a monkey kidney cell line (BGM) measured as the minimal lethal dose for 50% of cells (MDL_50_).

Molecule	MDL_50_ (µg/mL) Mean ± SD	W2		3D7	
		IC_50_ (µg/mL)* Mean ± SD	SI**	IC_50_ (µg/mL)* Mean ± SD	SI**
**LQB118**	≤3.9	0.17±0.1	23	0.16±0.03	24
**LQB192**	68.7±8.4	2.9±2.5	24	3.8±1	18
**LQB216**	24.2±3	0.4±0.2	60	0.21±0.09	115
**LQB221**	5.1	0.63±0.8	8	0.25±0.1	20
**LQB222**	≤3.9	1.8±1.6	2	3.5±0.4	1
**LQB223**	≥1000	10.2±1.2	98	7.8±3	128
**Chloroquine**	ND	0.138	-	0.03	-

The selectivity index (SI) is a ratio between toxicity and activity.

*IC_50_ ≤10 µg/mL are considered as active; 11–20 µg/mL as partially active (PA) and > 20 µg/mL as inactive.

**SI based in results from three experiments; values bellow 10 are indicative of drug toxicity. ND = not done.

Compounds LQB118, LQB192, LQB221 and LQB222, although active, were toxic to BGM cells; thus, in spite of their activity *in vitro*, they displayed low selectivity indexes (SI). Compound LQB223, in spite of being less active, showed higher SI (128 and 98), respectively, for the 3D7 and W2 parasites; the second best compound was LQB216, with SI of 115 and 60, respectively, for these parasite lines.

The most promising compound, LQB223, was then tested in mice infected with *P. berghei* malaria induced by blood forms. Given by gavage, in daily doses of 100 or 50 mg/kg, for three days, LQB223 reduced parasitemia, respectively, in 67% and 30% on the fifth day of infection (Table 2).

**Table 2:** *P. berghei* (NK65 chloroquine-sensitive strain) parasitemia and reduction at days 5 and 7 of infection, in mice treated with either LQB223 or chloroquine in relation to control non-treated mice.

Treatment with	Dose (mg/kg)	Mean Parasitemia ± SD (% Reduction)	
		5^yh^ Day	7^th^ Day
*Exp.1*			
**Control non-treated**	**0**	0.5±0.2	15±4.4
**Chloroquine**	**5**	0 (100%)	7.3±3 (52%)
**LQB223**	**100**	0.15±0.2 (67%)	11±3 (28%)
*Exp.2*			
**Control non-treated**	**0**	1.7±0.8	21±8
**Chloroquine**	**15**	0±0.0 (100%)	0±0.0 (100%)
**LQB223**	**50**	1.2±0.5 (30%)	18.3±1.5 (13%)

### Molecular dynamics

**Figure 4:**
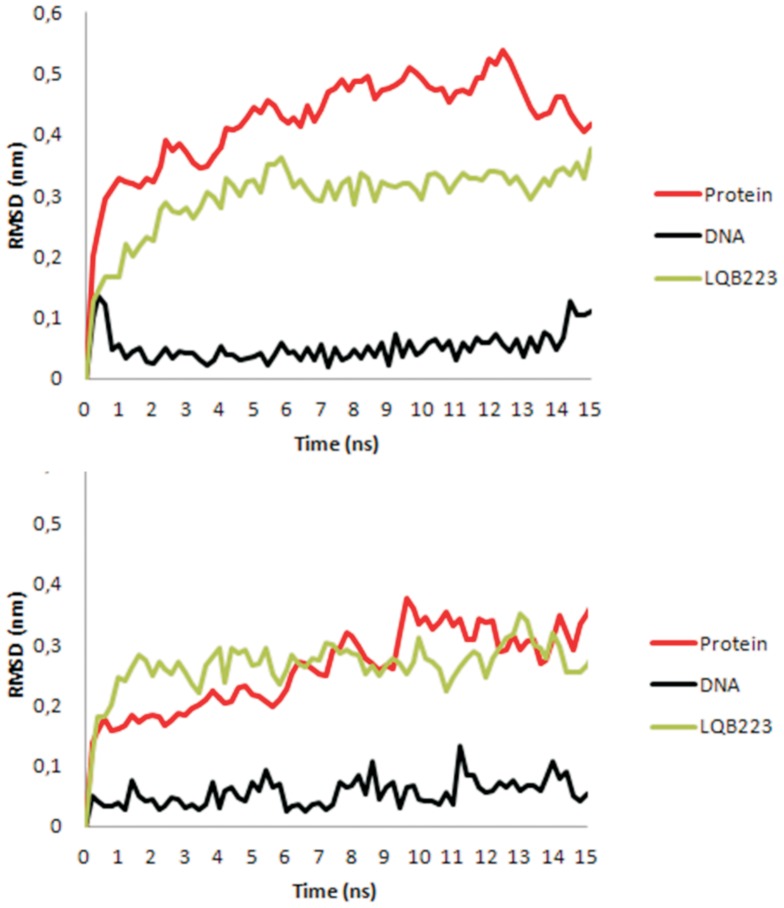
Temporal RMSD values for the LQB223, DNA an *Pf*TopoI (upper) an *Hss*RopoI (lower). These values were counted in the time range of 0–15,000 ps, at each 20 ps.

In order to validate the docking methodology and to understand the drug selectivity observed *in vitro* with LQB223, this promising compound was further studied considering the dynamic behavior inside the topotecan binding sites of *Hss*TopoI and *Pf*TopoI. The temporal RMSD calculations were carried out on all atoms of each complex to give frames every 200 ps during the 15 ns of simulation. Figure 4 shows the equilibration of each system during the first 1 ns.

The MD studies show that LQB223 presents distinct dynamical behavior inside *Pf*TopoI and *Hss*TopoI. Considering Lennard-Jones short-range (LJ-SR) and Coulombic short-range (Coul-SR) potential energy calculations between protein, DNA, and ligands, it was observed that LQB223 interacts differently inside *Pf*TopoI and *Hss*TopoI (Table 3). LQB223 displays relatively lower energy pattern inside *Pf*TopoI compared to *Hss*TopoI. The simulations also show that the interactions energies for the interaction between LQB223 and DNA are lower in the *Hss*TopoI binding site.

**Table 3:** Lennard-Jones energies short-range (LJ-SR) and Coulombic potential within R-coulomb Coul-SR) between LQB223, protein, DNA and water during MD simulations.

	*Hss*Topo1 (kJ mol^−1^)				*Pf*Topo1 (kJ mol^−1^)			
	Coul-SR	LJ-SR	Coul-SR + LJ-SR	Estimated Error	Coul-SR	LJ-SR	Coul-SR + LJ-SR	Estimated Error
**Protein-LQB223**	2.4	−29.8	−27.4	4.1	1	−42.4	−41.4	5.0
**DNA-LQB223**	−6.6	−144.3	−150.9	7.7	−4.95	−121.9	−126.8	6.5
**Water-LQB223**	−5.9	−59.61	−65.5	5.4	−5.68	−73.5	−79.1	7.1
**Total**			−243.8				−247.3	

## Discussion

Previous studies identified some flavonoids as DNA intercalators, *Hss*TopoI and *Pf*TopoI inhibitors, suggesting that topoisomerase I may be a molecular target for these compounds [40], [41]. All LQBs previously prepared presented antineoplasic [12], [41], [42] and antiparasitic [43], [44] properties *in vitro* [12], [42], [43], [44] and *in vivo* [44]. Considering that and their similarity with the two known Topo I inhibitors camptothecin and topotecan (Figure 1), they are expected to be potential inhibitors of *Pf*TopoI. The best way to provide this theory would be performing experimental analysis of the LQBs inside the *Pf*TopoI. As our group does not have access to the enzyme, we performed computational analysis to understand the dynamic of these compounds, which give us an idea of inhibition and toxicity. But, additional experimental work should be performed to validate *Pf*TopoI as a molecular target for the LQBs. The docking results suggest that the LQBs fit well into the binding sites of camptothecin and topotecan, although the absolute values were lower than those obtained with the inhibitors. The Topo I-DNA-drugs complex demonstrate that all LQBs intercalate at the site of DNA cleavage and are stabilized by π-stacking interactions (data not shown), suggesting they act as uncompetitive inhibitors, as previously reported for topotecan [17]. However, only LQB223 is likely to be potentially selective, considering the differences observed in the docking energies for this compound in *Pf*TopoI and *Hss*TopoI (Table S1). This molecule also presented the closest docking energies to topotecan and camptothecin. The location of LQB223 inside *Pf*TopoI suggests that the interactions with the residues Arg312 (11.4 Kcal mol^−1^), Asp513 (−6.0 Kcal mol^−1^), Gln698 (−11.5 Kcal mol^−1^), and DNA (−145.6 Kcal mol^−1^) are relevant for drug selectivity.

Most Topo I inhibitors tested showed a similar activity profile against *P. falciparum* parasites CQ-resistant (W2) or CQ-sensitive (3D7); however, only two compounds were promising as new antimalarial targets (LQB223 and LQB216), active against both strains, and displayed the highest therapeutic index, i.e., SI of 98 and 60 for W2, and 128 and 115 for 3D7. Compound LQB222 was the most toxic, with SI bellow 10 for both strains.

Data *in vitro* for LQB223, the only *N*-tosyl-azapterocarpan with the best SI value, corroborate the docking results, and explain the data *in vivo*, when it also inhibited malaria in mice infected with *P. berghei.*

MD results suggest that this molecule fits well in the binding site during the simulation, showing the system stabilization. The differences observed in the short-range potential energy calculations suggest a reasonable explanation for the selectivity of LQB223 for *Pf*TopoI over *Hss*TopoI observed in the experimental studies. The MD simulations showed that the energies of interaction between LQB223 and the protein are more favorable inside *Pf*TopoI, corroborating with the docking results. However, in contrast with the docking calculations, MD studies indicate that LQB223 interacts better with DNA in *Hss*TopoI instead of *Pf*TopoI. An analysis of the RMSF indicates that the fluctuation of the residues of *Pf*TopoI is higher than in *Hss*TopoI (Figure S2). This result is similar with previous published data showing that the residues in the plasmodial enzyme displays a larger flexibility than the human enzyme when docked with camptothecin [45] and raises the possibility of other molecular targets being responsible for the *in vitro* and *in vivo* activity observed for LQB223.

The search for new inhibitors of specific drug targets of *P. falciparum* has been explored as a promising way to develop new antimalarials [9], [46], [47]. The differences in the binding sites between *Pf*TopoI and *Hss*TopoI suggest this enzyme as a suitable selective target for the drug design against *P. falciparum*. According to the *in vitro* results, all LQBs are potential candidates to *Pf*TopoI inhibitors, being the LQB223 the most promising compound, considering that this molecule presented the highest SI and was also active *in vivo*. It suggests LQB223 as a promising lead compound to develop new antimalarials. The interactions with DNA and residues Arg312, Asp513 and Gln698 of *Pf*TopoI may be explored in further studies of new potential inhibitors of *Pf*TopoI.

## Supporting Information

Figure S1 **3D protein model of** ***Pf*** **TopoI.**
(TIF)

Figure S2 **Per-residue Root Mean Square Fluctuations (RMSF) of the** ***Hss*** **Topo1 and** ***Pf*** **TopoI.**
(TIF)

Table S1 **Interaction energies between the LQBs, DNA and the residues of** ***Hss*** **TopoI and** ***Pf*** **TopoI.**
(PDF)
