# Supplementary figures and images for: Theoretical and Experimental Studies of New Modified Isoflavonoids as Potential Inhibitors of Topoisomerase I from Plasmodium falciparum
Source: PLoS One. 2014 Mar 20;9(3):e91191. doi: 10.1371/journal.pone.0091191 (PMC3961230; doi:10.1371/journal.pone.0091191)

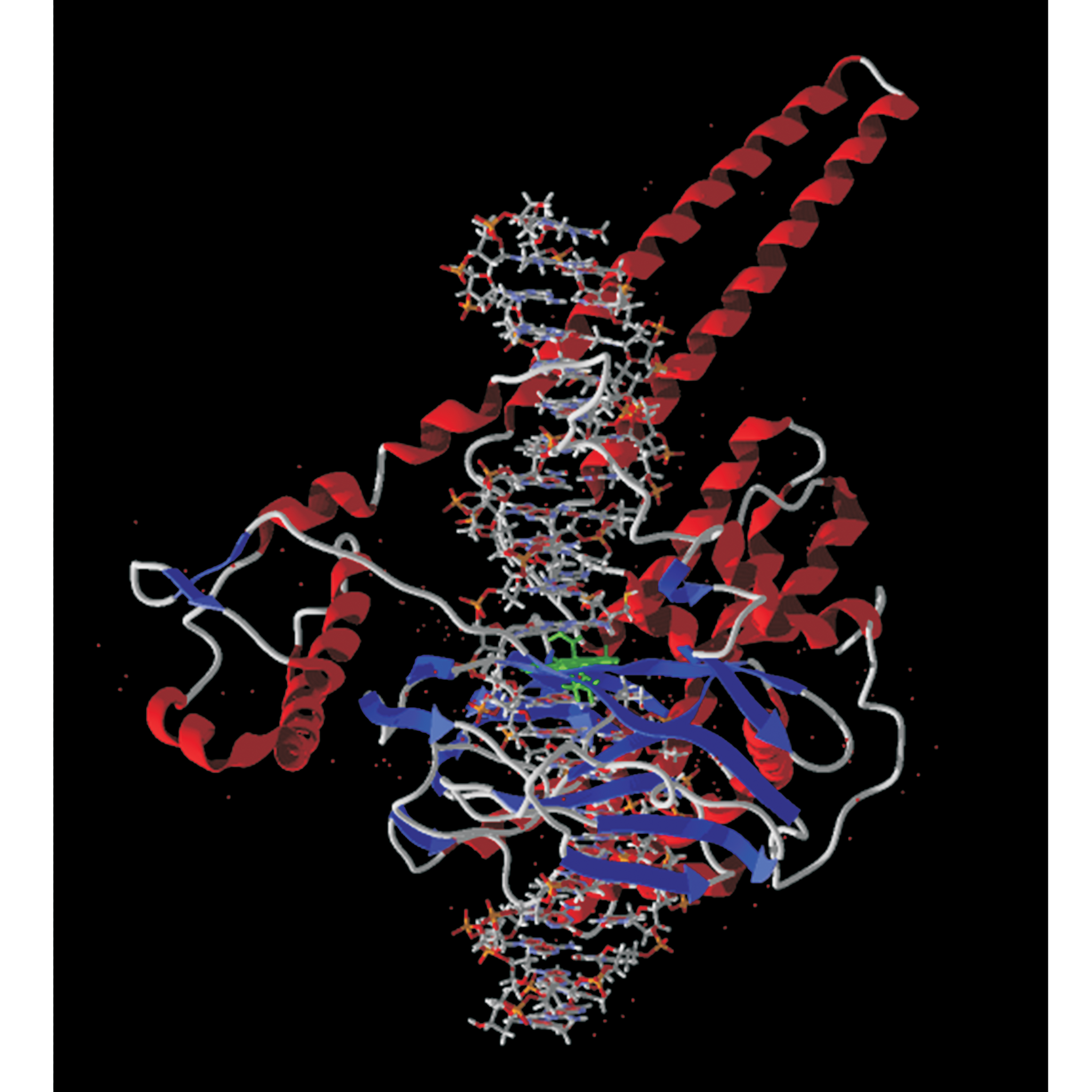

Supplement: Figure S1 — 3D protein model of Pf TopoI. (TIF) [file pone.0091191.s001.tif]

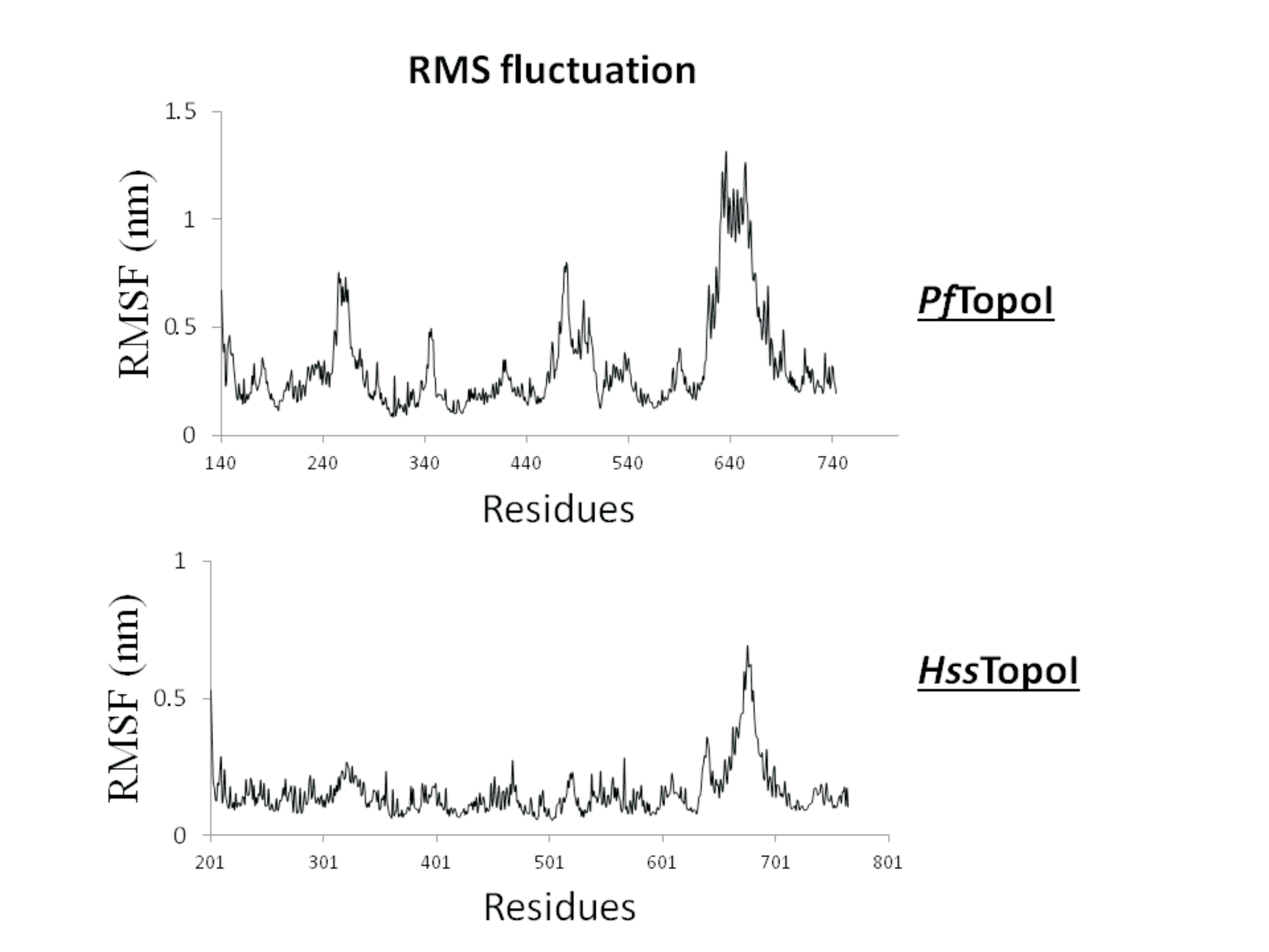

Supplement: Figure S2 — Per-residue Root Mean Square Fluctuations (RMSF) of the Hss Topo1 and Pf TopoI. (TIF) [file pone.0091191.s002.tif]
