# Supplementary material for: Theoretical and Experimental Studies of New Modified Isoflavonoids as Potential Inhibitors of Topoisomerase I from Plasmodium falciparum
Source: PLoS One. 2014 Mar 20;9(3):e91191. doi: 10.1371/journal.pone.0091191 (PMC3961230; doi:10.1371/journal.pone.0091191)
Supplement: Table S1 — Interaction energies between the LQBs, DNA and the residues of Hss TopoI and Pf TopoI. (PDF) [file pone.0091191.s003.pdf]

Supplementary Material

Table S1. Interaction energies between the LQBs, DNA and the residues of *Hss*TopoI and *Pf*TopoI.

**Compound**	***Hss*Topo1**			***Pf*Topo1**		
	**Total Energy (kcal mol^{-1})**	**Residue**	**Energy (kcal mol^{-1})**	**Total Energy (kcal mol^{-1})**	**Residue**	**Energy (kcal mol^{-1})**
Topotecan	-237.5	DNA	-186.4	-238.6	DNA	-187.9
		Asp533	-10.7		Asp513	-10.4
		Arg364	-8.6		Glu304	-8.8
		Glu356	-5.8		Arg312	-8.3
		Thr718	-3.9		Gln698	-7.1
		Asn352	-3.8		Lys512	-3.4
Camptothecin	-211.1	DNA	-175.7	-212.7	DNA	-175.6
		Asp533	-9.6		Asp513	-10.0
		Arg364	-9.3		Arg312	-9.0
		Lys532	-4.9		Gln698	-7.1
		Thr718	-4.5		Lys512	-4.8
		Asn722	-1.4		Glu304	-0.8
LQB192	-95.6	DNA	-92.5	-96.4	DNA	-91.9
		Lys425	-9.9		Arg312	-9.9
		Trp416	-5.7		Asp513	-6.2
		Asn352	-5.0		Gln698	-5.0
		Tyr426	-4.4		Lys512	-2.0
		Arg364	-2.4		Ile515	-0.8
LQB223	-171.2	DNA	-132.5	-187.5	DNA	-165.3
		Asn352	-9.3		Gln698	-11.5
		Arg364	-4.3		Arg312	-11.4
		Glu356	-4.1		Asp513	-6.0
		Tyr452	-3.8		Lys512	-2.5
		Lys425	-3.0		Thr701	-1.8
LQB118	-144.4	DNA	-133.2	-143.4	DNA	-133.6
		Arg364	-10.2		Arg312	-10.1
		Asp533	-6.9		Asp513	-6.8
		Asn722	-2.8		Lys512	-2.5
		Lys532	-2.5		Gln698	-2.4
		Thr718	2.0		Thr702	-0.5

LQB221	-160.0	DNA	-121.6	-156.2	DNA	-122.2
		Lys425	-13.9		Lys376	-12.5
		Tyr426	-5.7		Tyr377	-6.4
		Asn352	-4.9		Tyr367	-6.3
		Glu356	-4.3		Arg312	-4.0
		Trp416	-4.1		Glu304	-3.8
LQB222	-154.4	DNA	136.3	-155.4	DNA	-136.8
		Arg364	-11.9		Arg312	-11.6
		Asp533	-7.0		Asp513	-6.7
		Lys532	-3.4		Glu698	-4.4
		Thr718	-3.3		Lys512	-3.5
		Ile535	-1.4		Ile695	-2.1
LQB216	-146.9	DNA	-134.1	-145.8	DNA	-133.6
		Lys425	-6.3		Lys376	-6.7
		Glu356	-4.1		Glu304	-3.9
		Arg364	-3.9		Arg312	-3.3
		Glu418	-3.0		Asp369	-3.0
		Phe361	-0.5		Asn372	-0.5
